# Sequential catalytic lignin valorization and bioethanol production: an integrated biorefinery strategy

**DOI:** 10.1186/s13068-024-02459-8

**Published:** 2024-01-20

**Authors:** Yilu Wu, Changsheng Su, Zicheng Liao, Gege Zhang, Yongjie Jiang, Yankun Wang, Changwei Zhang, Di Cai, Peiyong Qin, Tianwei Tan

**Affiliations:** 1 National Energy R&D Center for Biorefinery, Beijing University of Chemical Technology, Beijing, 100029 People’s Republic of China; 2 College of Life Science and Technology, Beijing University of Chemical Technology, Beijing, 100029 People’s Republic of China; 3 School of International Education, Beijing University of Chemical Technology, Beijing, 100029 People’s Republic of China

**Keywords:** Reductive catalytic fractionation, Poplar sawdust, *Saccharomyces cerevisiae*, Bioethanol, Fermentation

## Abstract

**Background:**

The effective valorization of lignin and carbohydrates in lignocellulose matrix under the concept of biorefinery is a primary strategy to produce sustainable chemicals and fuels. Based on the reductive catalytic fractionation (RCF), lignin in lignocelluloses can be depolymerized into viscous oils, while the highly delignified pulps with high polysaccharides retention can be transformed into various chemicals.

**Results:**

A biorefinery paradigm for sequentially valorization of the main components in poplar sawdust was constructed. In this process, the well-defined low-molecular-weight phenols and bioethanol were co-generated by tandem chemo-catalysis in the RCF stage and bio-catalysis in fermentation stage. In the RCF stage, hydrogen transfer reactions were conducted in one-pot process using Raney Ni as catalyst, while the isopropanol (2-PrOH) in the initial liquor was served as a hydrogen donor and the solvent for lignin dissolution. Results indicated the proportion of the 2-PrOH in the initial liquor of RCF influenced the chemical constitution and yield of the lignin oil, which also affected the characteristics of the pulps and the following bioethanol production. A 67.48 ± 0.44% delignification with 20.65 ± 0.31% of monolignols yield were realized when the 2-PrOH:H_2_O ratio in initial liquor was 7:3 (6.67 wt% of the catalyst loading, 200 °C for 3 h). The RCF pulp had higher carbohydrates retention (57.96 ± 2.78 wt%), which was converted to 21.61 ± 0.62 g/L of bioethanol with a yield of 0.429 ± 0.010 g/g in fermentation using an engineered *S. cerevisiae* strain. Based on the mass balance analysis, 104.4 g of ethanol and 206.5 g of lignin oil can be produced from 1000 g of the raw poplar sawdust.

**Conclusions:**

The main chemical components in poplar sawdust can be effectively transformed into lignin oil and bioethanol. The attractive results from the biorefinery process exhibit great promise for the production of valuable biofuels and chemicals from abundant lignocellulosic materials.

**Supplementary Information:**

The online version contains supplementary material available at 10.1186/s13068-024-02459-8.

## Background

In light of the environmental pollutions caused by massive usage of unsustainable fossil fuels, there is an increasingly interests in lignocellulosic bioethanol production [1]. However, several technical barriers in the production chain of renewable cellulosic ethanol still hinder the industrialization [2, 3]. For instance, one urgent R&D priority for bioethanol production is the development of alternative techniques to effectively overcome the recalcitrant structure of the complex lignocellulosic matrix, thereby releasing locked polysaccharides for subsequent enzymatic hydrolysis into monomeric sugars [4]. To this end, a primary strategy is to adopt a proper lignocellulose fractionation process to expose the cellulose and hemicellulose components [5]. Over the past decades, a variety of biomass fractionation techniques, including the physical, chemical, and biological processes, have been suggested to decompose the lignocellulose structure [6]. Nonetheless, most existing lignocellulosic fractionations are exclusively focused on carbohydrates production, where the lignin fraction, a complex phenolic biopolymer, is often regarded as an inferior energy source for burning [7].

In recent years, reductive catalytic fractionation (RCF) has been regarded as a promising approach for lignocellulose decomposition under the concept of lignin-first biorefinery. In this process, a high-quality pulp can be obtained accompanied with the generation of valuable monolignols-enriched lignin oil [8–10]. In a typical RCF process, heterogeneous transition metal catalysts (e.g., Ni/C, Pd/C, Ru/C, etc.) and hydrogen donors (commonly using the H_2_ or the organic solvents in hydrogen transition process, e.g., the 2-PrOH and methanol) are involved in a solvent pulping process, which can accelerate the highly selective cleavage of the inner linkages in native lignin and avoid the condensation/repolymerization of the lignin fragments [11–13]. At the same time, the presence of the catalysts in the RCF processes could directly affect the pH level of the soluble fractions, thus reducing the auto-hydrolysis of hemicelluloses and leading to high retention of carbohydrates in pulp [11, 14, 15].

Previous literature studies suggested that the retention of carbohydrates and delignification in RCF pulping were higher than that of organosolv pulping without the assistance of catalysts and hydrogen [16, 17]. As for the downstream valorization of the RCF streams, the passivated lignin fractions in the RCF oil, bearing different end-chains, are amenable as the starting molecules for transformation into valuable chemicals and materials [18–20]. For instance, monolignols can be further derived into phenols, aviation fuels, and medicines [21–23], while the lignin oligomers from RCF oil could be used to replace unsustainable petrol-based polyols for the fabrication of polyurethanes [20, 24]. In another study, Ebikade et al. used crude lignin oil as antimicrobials against *Staphylococcus aureus* [25]. The RCF pulps can be either catalyzed into furan derivatives and sugar alcohols, or enzymatically hydrolyzed into monomeric sugars for subsequent fermentative transformations [26–28].

However, compared with the rapidly developed catalytic valorizations of lignin oil, fermentative valorization of the RCF pulps are still rarely reported in previous reports [29]. Van den Bosch et al. [30] used a Ni-Al_2_O_3_ catalyst to integrate lignin valorization and bioethanol fermentation in a RCF process of birch powder. After 120 h of semi-simultaneous saccharification and fermentation (S-SSF) by *S. cerevisiae* GSE16-T18-HAA1*, an ethanol titer of 36 g/L (73% of the theoretical yield) was obtained. In another work, Wu et al. revealed that the residual RCF oil remaining on the surface of poplar pulps leaded to the severe inhibition of microorganisms, and an ethanol yield of 0.45 g/g (monomeric sugars in hydrolysate) with 17.77 g/L of ethanol can be achieved under separate hydrolysis and fermentation (SHF) in batch mode [31].

In fact, the economic feasibility of the RCF process can be significantly improved, because bioethanol can be co-generated from the fractionated pulps [29]. Technol-economic analysis reflected that the production of bioethanol was expected to offset the total cost of the lignin oil production by $ 0.75/kg, and the catalytic upgrading of bioethanol for longer lifetime applications would further increase the environmental benefits [32]. Therefore, in a scheme of ‘zero waste’ biorefinery, aiming to improve the applicability of the RCF process, it is necessary to continuously strengthen the fermentative bioethanol production from RCF pulps by process engineering.

Herein, aiming to sequentially valorize the lignin and carbohydrate fractions in lignocellulose, the RCF and fermentation processes were cascaded, providing an economically strategy for lignin oil and bioethanol productions from dry poplar sawdust. The early-stage conversion of lignin into stable oligomers and monomers was realized by the action of Raney Ni as hydrogenation catalyst, while the 2-PrOH was served as the hydrogen donor and solvent. A systematic study on the effect of 2-PrOH proportion in the initial liquor was conducted, and the lignin oil and pulps were analyzed, followed by conducting bioethanol production using an engineered strain. The biorefinery process provided an efficient pathway in transformation of the lignocellulose matrix into well-defined biofuels and chemicals.

## Results

### Chemical composition of the RCF pulps

The lignin oil and pulp obtained by early-stage hydrogen transfer fractionation of poplar sawdust was conducted using Raney Ni catalyst and a 2-PrOH–H_2_O mixture as the initial liquor. In this process, the 2-PrOH was not only served as the solvent for lignin dissolution, but also used as a hydrogen donor. Herein, in the RCF process, different 2-PrOH:H_2_O ratios in the initial liquor on the lignin oil and the pulp production were investigated (Additional file 1: Table S1). Generally, the yield and chemical constitution of the lignin oils were influenced by the 2-PrOH proportion. An increase in the 2-PrOH ratio in the initial liquor led to the increased lignin oil yield (Additional file 1: Table S2). This phenomenon was consistent with the results in a previous work [15], which was ascribed to the an excessive amount of the hydrogen donor that guaranteed sufficient hydrogenation of the lignin fragments in the initial liquor with higher 2-PrOH ratio [16]. Besides, the Hildebrand solubility parameter (*δ*_*T*_) (HSP) of the initial liquors with higher 2-PrOH proportions, which are similar to the lignin fractions in poplar sawdust, could be another reason for the high delignification (Additional file 1: Table S3; Additional file 1: Fig. S1). A closely matched HSP would promote the delignification of the lignocellulose matrix [33–35] (Table 1).

**Table 1:** Component analysis of the RCF pulps fractionated by different ratios of 2-PrOH:H_2_O

2-PrOH:H_2_O ratios (v/v)	Solid recovery (wt%)	Glucan (wt%)	Xylan (wt%)	Acid-soluble lignin (wt%)	Acid-insoluble lignin (wt%)	Glucan recovery (%)	Xylan recovery (%)	Delignification (%)
1:9	47.85 ± 1.25	73.06 ± 4.98	1.01 ± 0.084	3.55 ± 0.11	15.33 ± 0.23	98.63 ± 6.72	1.37 ± 0.114	65.99 ± 0.78
3:7	47.91 ± 0.46	79.66 ± 2.55	2.77 ± 0.084	4.99 ± 0.13	9.00 ± 0.31	96.64 ± 3.09	3.36 ± 0.102	81.62 ± 0.54
5:5	49.94 ± 0.98	75.88 ± 5.05	4.99 ± 0.066	6.60 ± 0.10	8.00 ± 0.11	93.83 ± 6.24	6.17 ± 0.082	82.60 ± 0.32
7:3	57.96 ± 2.78	72.67 ± 5.46	5.01 ± 0.32	6.99 ± 0.24	14.33 ± 0.16	93.63 ± 7.03	6.37 ± 0.407	67.48 ± 0.44
9:1	53.98 ± 2.11	71.10 ± 1.84	2.15 ± 0.44	4.22 ± 0.27	16.00 ± 0.20	97.06 ± 2.51	2.94 ± 0.602	53.98 ± 0.57

### Characterizations of the reductive catalytic fractionated lignin oil

**Fig. 1:**
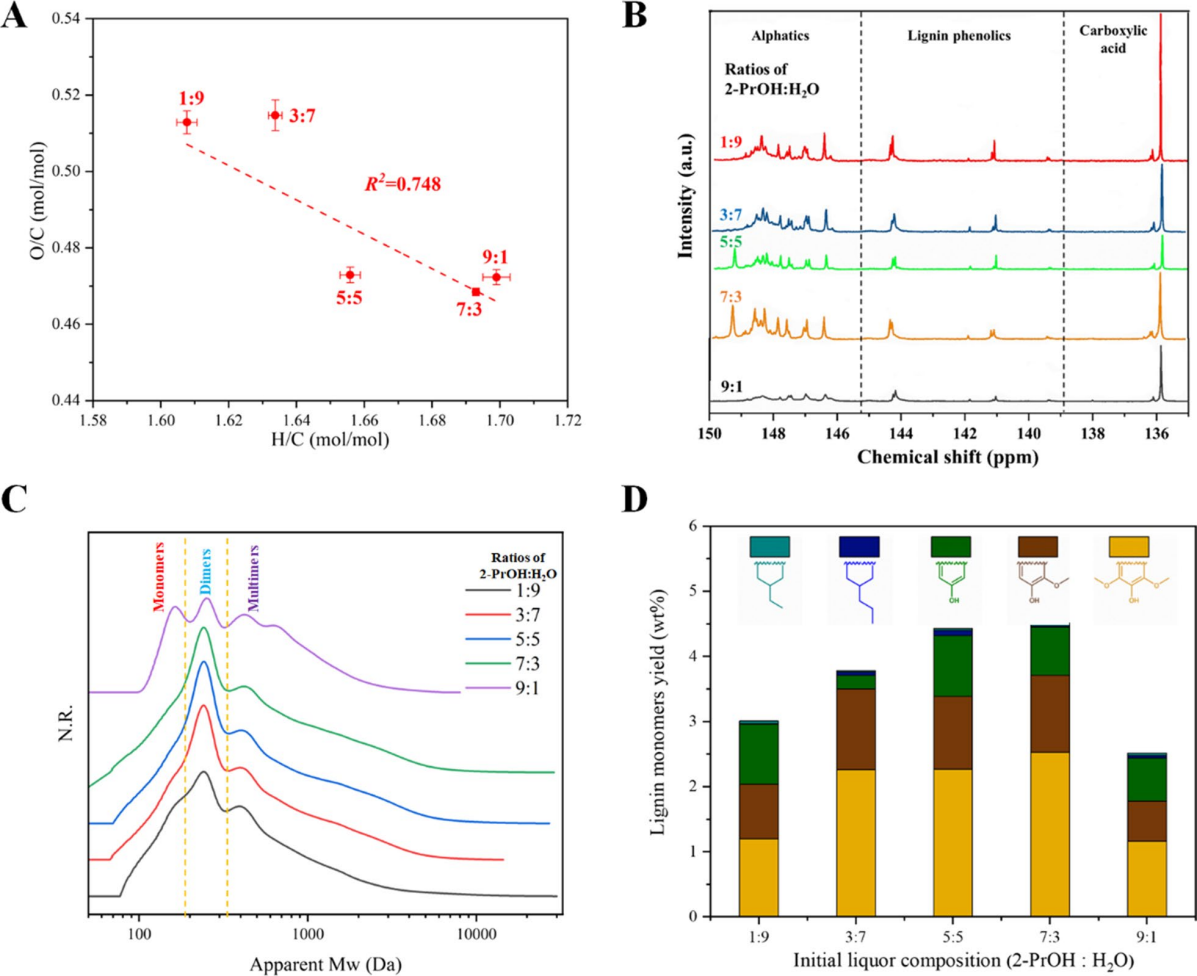
**a** Plot of H/C ratio versus O/C ratio of the lignin oil obtained by RCF with different ratios of 2-PrOH:H_2_O (v/v) in initial liquor. **b** ^31^P-NMR spectrums of the lignin oil. **c** Molecular weight distribution, and **d** the lignin monomers yield of the RCF oil with different ratios of 2-PrOH/H_2_O

To better understand the impact of 2-PrOH proportion in the initial liquor on the reductive reactions in the RCF process, O/C and H/C molar ratios in the lignin oils were analyzed (Fig. 1a). Elemental analyses showed that the molar ratios of O/C and H/C in lignin oils after RCF varied with the 2-PrOH ratio in the initial liquor (O/C ratio decreased and H/C ratio increased with the increase 2-PrOH ratio in initial liquor). When the 2-PrOH content is lower, the effect of hydrodeoxygenation became pronounced as the proportion of the hydrogen donor in the system was reduced and the γ-OH group of lignin became additional hydrogen donor. In contrast, hydrogenation became the main reductive reaction with the increase of 2-PrOH proportion in the initial liquor.

The molecular weight distributions of lignin oils are shown in Fig. 1c. Regardless of the proportion of 2-PrOH in the initial liquor, a narrow molecule weight distribution of the lignin oils could be realized, and the *Mw* of the specimens were concentrated in the range of 100–600 Da. We speculated that the relatively low molecule weight of the lignin oil was ascribed to excessive breaking of the β-O-4 bond during lignin depolymerization, and the inhibition of the condensation by reductive stabilization [36]. Meanwhile, the molecular weight distribution illustrated that with the increase of 2-PrOH proportion in the initial liquor, the weight average molecular weight (*Mw*) and number average molecular weight (*Mn*) was gradually increased (Fig. 1c; Additional file 1: Table S4). However, when the 2-PrOH proportion was higher than 70%, the average molecular weight decreased. This was attributed to the co-generation of a small amount of sugar polyols from cellulose and hemicellulose [17].

The monolignols content in the oil was quantitatively analyzed. Generally, the monomeric lignins in the specimens can be classified into G-, S-, and H-units, as well as cyclohexanols. The quantitatively calculated results are shown in Additional file 1: Table S3. It was confirmed that the hydrogenation, hydrogenolysis and dehydrogenation of lignin were involved in the RCF process simultaneously [37], and the initial liquor with 70% of 2-PrOH exhibited the highest lignin monomers yield of 4.52 wt%.

Literatures showed that the hydrogenation of aromatic ring became less favorable in the absence of a hydrogen donor [38, 39]. We hypothesized that a lower hydrogen donor content in the RCF process led to a decreased hydrogenation effect on the aromatic ring, and this hypothesis can be clarified from Fig. 1d. Other reactive pathway was the selective production of alkyl-substituted phenols, caused by the interaction of the Raney Ni surface with the aliphatic OH group, the aromatic ring and the phenolic -OH of the lignin intermediates [38]. It is noteworthy that the yield of S- and G-units were the dominant types (Fig. 1d), and the S-units was increased when the proportion of 2-PrOH in initial liquor increased. However, demethylation was found to be more active in groups with lower levels of hydrogen donor in the initial liquor. The propyl group on the side chain of lignin monomers became shortened and tended to converted into ethyl and methyl ligninol derivatives [15]. Nonetheless, when the 2-PrOH:H_2_O ratio was 9:1 (v/v), the yield of lignin monomers decreased significantly to 2.52 wt%.

^31^P-NMR spectrums were performed to determine the hydroxyl content in the lignin oil, and three types of hydroxyl groups were identified. The order for the hydroxyl groups content was: aliphatic-OH > phenolic-OH > carboxy-OH, irrespective of the initial liquor composition (Additional file 1: Table S4). Generally, the aliphatic-OH content of lignin oil increased gradually with the increase of 2-PrOH content in the initial liquor. In the group with 2-PrOH:H_2_O ratio of 7:3 in initial liquor, the maximum aliphatic hydroxyl group content was realized at 31.72 mmol/g. Besides, the main structural unit in lignin oligomers was the G-monomers, rather than the S-monomers found in native poplar lignin, confirming that the demethylation of lignin was associated with the RCF process [40]. The relatively low content of carboxy-OH groups in the lignin oil was ascribed to the reduction-catalyzed reactions in the RCF process (Fig. 1b).

**Fig. 2:**
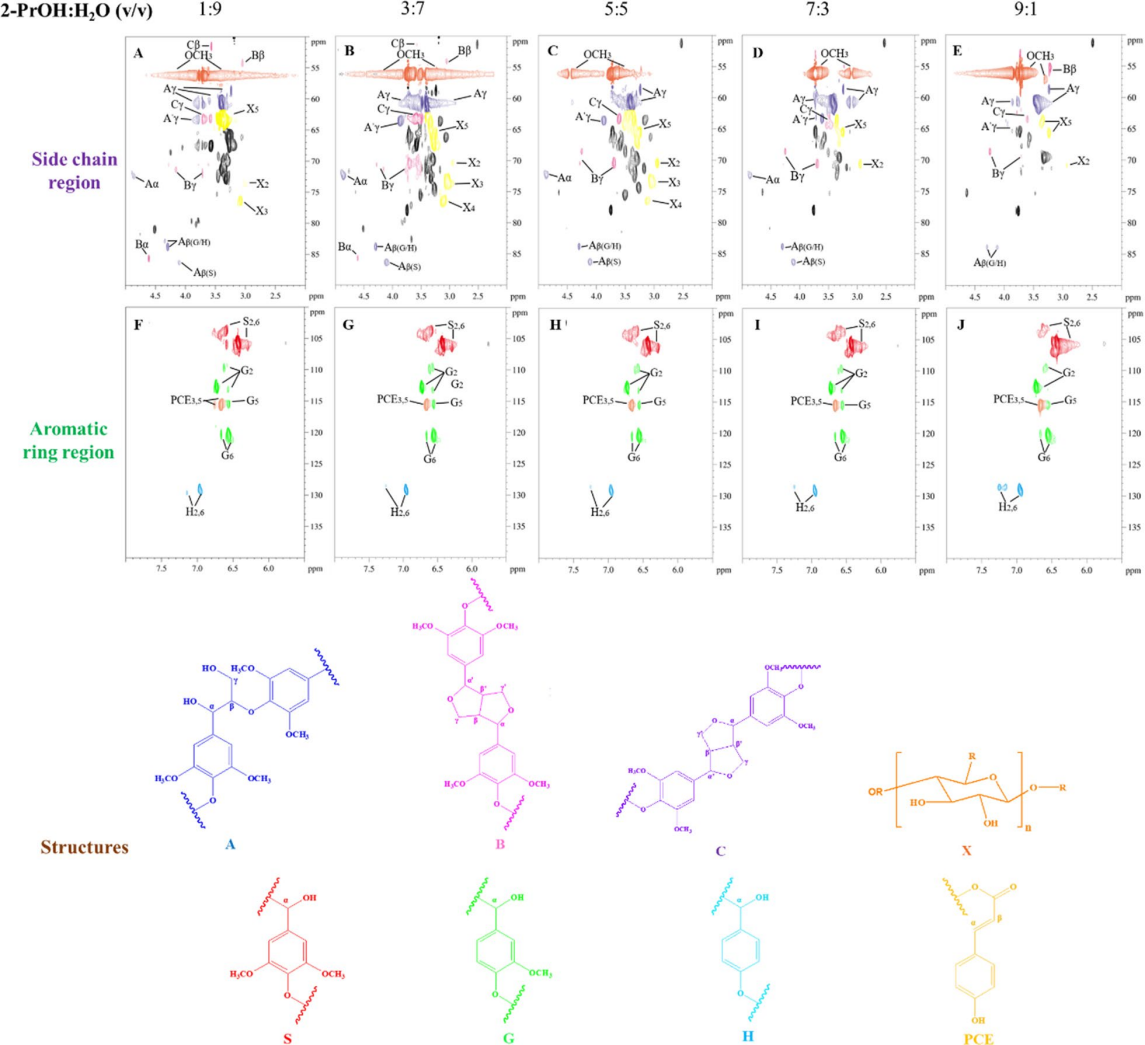
2D HSQC NMR spectrums of the lignin oil fractionated by different proportion of 2-PrOH:H_2_O (v/v) in the initial liquor. **a–e** The side chain region, and **f–j** the aromatic ring region of the lignin oil specimens

The 2D HSQC NMR spectra were further examined, with the major contours residing in the δ_C_/δ_H_ 50—90/2.0–5.0 ppm and δ_C_/δ_H_ 100–140/5.5–8 ppm regions (Fig. 2). The results of the semi-quantitative analysis of relative content for the 2D-HSQC are shown in Table 2. Generally, all the lignin oils contained residual β-O-4 bonds, and β–β and β-5 bond. The lowest β-O-4 content was found in the lignin oil corresponding to a 2-PrOH:H_2_O ratio of 7:3 (v/v) in the initial liquor, indicating excessive reductive cleavage of the ether bonds [41, 42]. In the aromatic ring region, the signals of S, G and H units were observed, while *p*-coumarate (PCE) unit had a lower abundance. In agreement with the conclusions obtained from monolignols analysis, the predominant monomer types in the lignin oil specimens were the S/G-type monomers. With the increase in the proportion of 2-PrOH in initial liquor, the demethylation in the RCF process intensified [15, 17]. As a result, the relative proportions of H-unit and G-unit increased, reaching a maximum of 9.34% for the H-unit and a minimum of 12.90% for the S-unit at a 9:1 (v/v) of 2-PrOH/H_2_O ratio.

**Table 2:** Semi-quantitative analysis of 2D-HSQC of the RCF oil using different ratios of 2-PrOH:H_2_O (v/v) as initial liquor

2-PrOH:H_2_O ratios (v/v)	S (%)	G (%)	H (%)	S/G	β-O-4	β-β	β-5
1:9	22.898	71.689	5.412	2.941	6.649	0.292	0.666
3:7	24.078	70.903	5.019	2.378	5.389	0.525	0.025
5:5	23.914	70.179	5.907	2.420	7.447	0.517	0.000
7:3	24.557	69.141	6.302	2.800	5.014	0.482	0.000
9:1	12.903	77.758	9.340	1.661	6.798	0.748	0.000

### Properties of the reductive catalytic fractionated pulps

The chemical components of the RCF pulps were determined, which helped to understand the depolymerization of lignin in poplar sawdust during the RCF process [43]. The chemical composition of the RCF pulps fractionated by the liquors with different 2-PrOH:H_2_O ratios is shown in Table 1. The results showed that the glucan content in the pulps were much higher than that of xylan. This was attributed to the fact that some organic acids was released from poplar sawdust in the RCF process, which facilitated the hydrolysis of hemicellulose [44]. Cellulose, on the other hand, was difficult to degrade in the RCF process [45]. The influences of the 2-PrOH:H_2_O ratios (v/v) on the chemical composition the RCF pulps were presented as follows. When the water content is high, the released sugars in the pulping liquor could serve as additional hydrogen donors and be oxidized into saccharic acids. This will increase the acidity of the pulping liquor, and promote the hydrolysis of hemicelluloses fractions [46]. In contrast, the 2-PrOH-dominated liquor would enhance the hydrogenation of sugars and transform them into sugars alcohols. The effectiveness of delignification is dependent on the polarity of the liquor, as shown in Additional file 1: Fig. S1.

**Fig. 3:**
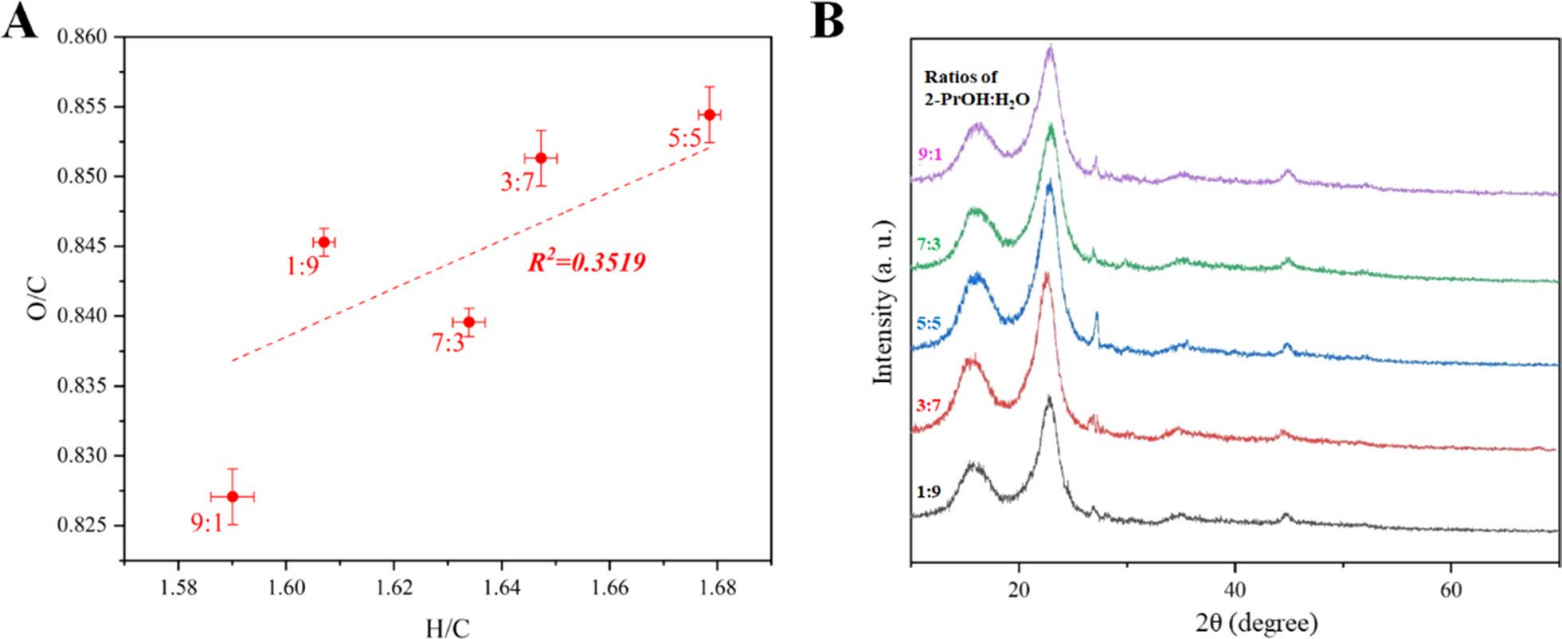
Characterizations of the RCF pulps using the initial liquor with different 2-PrOH/H_2_O (v/v) proportions. **a** Plot of H/C and O/C, and **b** X-ray diffraction spectrum analysis

The O/C and H/C ratios in all of the pulp samples increased after RCF due to delignification (Fig. 3a). XRD was used to analyze the crystallinity of the pulps (Fig. 3b; Additional file 1: Table S7). The increase of the 2-PrOH proportion in the initial liquor led to an increase of the crystallinity of the pulps. Compared with other groups, the 2-PrOH:H_2_O ratio of 7:3 (v/v) in initial liquor led to a maximal CrI of 66.5% which infers the effective removal of amorphous hemicellulose and delignification [47].

SEM was performed to compare the changes in morphology of the poplar sawdust samples before and after RCF (Additional file 1: Fig. S2). It revealed that the surface morphology of the poplar sawdust pulp changed significantly after the RCF. Different degrees of fiber separation appeared after RCF, and the increase in 2-PrOH proportion in the initial liquor caused more filamentous fibers. In addition, microporous structures appeared on the pulps’ surface (labeled red in high magnification SEM images), which initially appeared when the 2-PrOH:H_2_O ratio was 5:5 (v/v), and the microporosity increased with the increase of 2-PrOH proportion. This can be attributed to the migration of the lignin during the depolymerization [48, 49].

Saccharification of the RCF pulps was further investigated. The enzymatic hydrolysis of the RCF pulps was conducted under solid loading of 6 wt% and 12 wt% (Additional file 1: Table S1). The results showed that the sugars yields were maintained at a considerably high level irrespective of the solid loading. Compared to other groups, the pulps fractionated by the 3:7 (v/v) and 5:5 (v/v) liquor yielded higher sugar concentrations of 60.90 ± 0.39 g/L (55.20 ± 0.38 g/L glucose and 5.70 ± 0.33 g/L xylose) and 63.45 ± 0.67 g/L (55.80 ± 1.03 g/L glucose and 7.65 ± 0.64 g/L xylose), respectively.

### Bioethanol production using the engineered *S. cerevisiae* and the RCF pulps’ hydrolysates

As aforementioned, relatively high retention of carbohydrates fractions in the raw poplar sawdust were realized in all the RCF pulps. In addition, after enzymatic hydrolysis of the RCF pulps, xylose accounted for a large part of the overall monomeric sugars in the hydrolysates. Moreover, to achieve a high bioethanol yield from the RCF pulps, it is crucial to establish an effective xylose-assimilating strain. Generally, two primarily heterologous pathways were proposed in previous researches for xylose assimilation [50–52]. One is the XR-XDH pathway that heterologous expressed fungal-derived xylose reductase (XR) and xylitol dehydrogenase (XDH) [53]. In this process, the affinity of XR to nicotinamide adenine dinucleotide phosphate (NADPH) was significantly higher than that to nicotinamide adenine dinucleotide (NADH), however, the XDH needed to use NAD^+^ as a cofactor. Consequently, the imbalanced cofactors limited the utilization of pentose by the engineered strains [54, 55]. In our previous work, *S. cerevisiae* YSX4C222 was constructed with the plasmids of PRK-Rubisco module (CO_2_ fixation pathway) and the mXR-XDH module (the pathway contained a XR, a XDH, a XK and a NADH-preferred XR(R276H) mutants) by rational metabolic engineering [56]. This strain could balance the cofactors metabolism in the cells, which was able to consume high-level mixed sugars (70 g/L maltose and 40 g/L xylose) and achieve a total sugar consumption rate of 3.1 g/L h with ethanol yield of 0.47 g/g in synthetic medium.

In the current work, an engineered *S. cerevisiae* strain YL23-1 capable of efficient xylose assimilation was constructed by heterologous expressing the mXR-XDH pathways into genome, followed by integrating the putative high-affinity xylose transporter-associated genes *XUT4* and *XUT6* from *S. stipitis* [57, 58], and the adaptive laboratory evolution (ALE) screening. The fermentation performances of 4 candidate strains, including the parent strain M3013, the strain YL13 that expressed mXR-XDH pathways, the strain YL23 that further integrated transporter-associated genes, and the strain YL23-1 that was screened by ALE of YL23 using synthetic medium containing xylose as solo carbon source, were compared (Additional file 1: Table S8; Additional file 1: Fig. S3). As expected, attributed to robust xylose consumption after xylose transport enhancement and evolution, the evolved engineered strain YL23-1 exhibited a better cell growth rate. Meanwhile, the xylose consumption and ethanol productivity of the strain YL23-1 were also higher than the parent and other engineered strains (14.77 ± 0.60 g/L of ethanol can be produced from 40 g/L of xylose within 72 h of inoculation of the strain YL23-1).

**Fig. 4:**
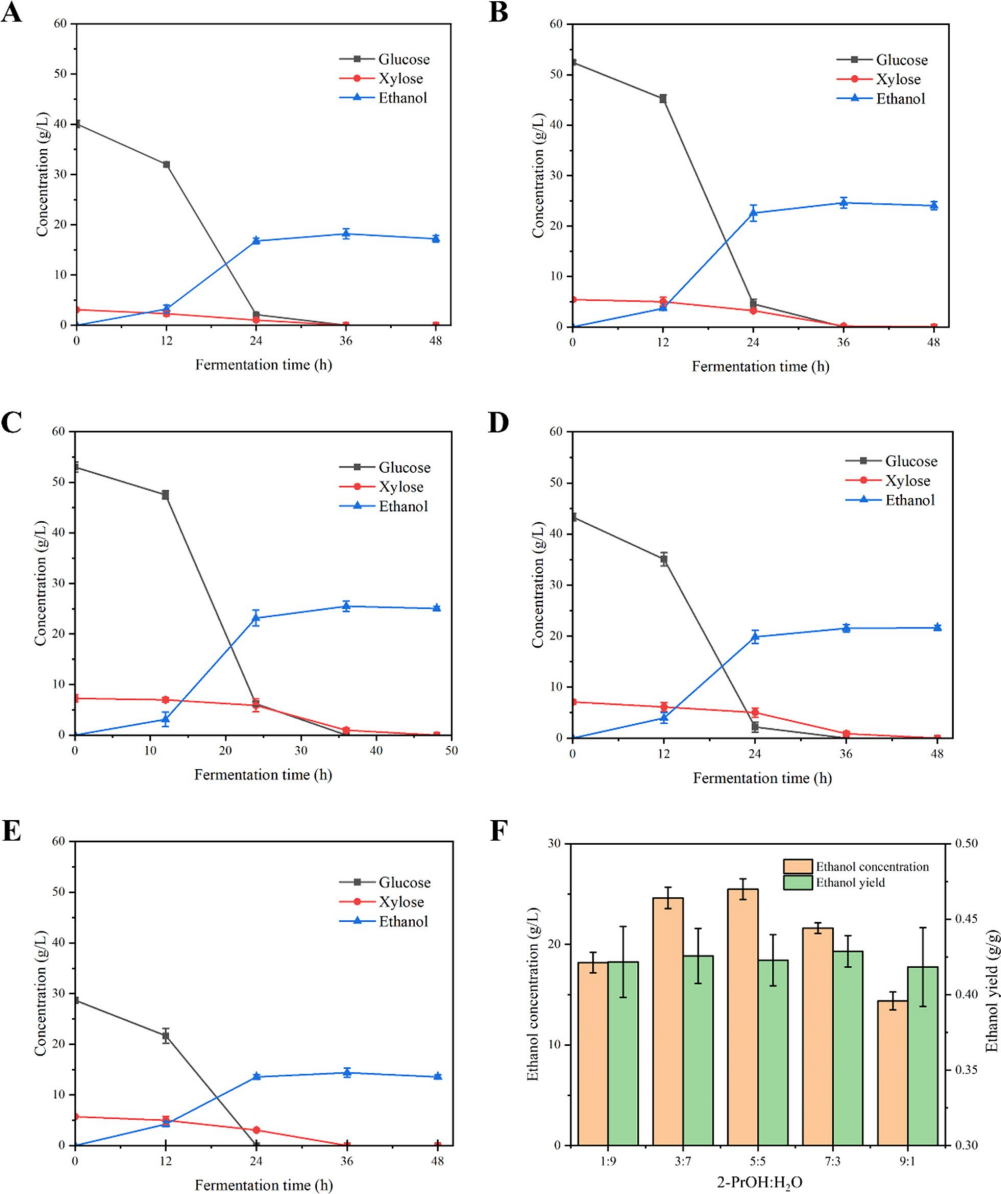
Bioethanol fermentation performances of *S. cerevisiae* YL23-1 by RCF pulps’ hydrolysates with 12 wt% of solid loading. The effect of the initial liquor proportion of 2-PrOH:H_2_O (v/v) on bioethanol production were analyzed. Bioethanol fermentation performances using the RCF pulps’ hydrolysates from the groups of **a** 1:9 (v/v), **b** 3:7 (v/v), **c** 5:5 (v/v), **d** 7:3 (v/v), and **e** 9:1 (v/v) of 2-PrOH:H_2_O. **f** Comparison of the bioethanol concentration and yield in different groups

The attractive xylose metabolism performances of strain YL23-1 prompted us to further use the hexose and pentose enriched RCF pulps’ hydrolysates as the substrate for bioethanol production, and the fermentation performances were investigated. Figure 4 shows all the monomeric sugars in the RCF pulps’ hydrolysates with 12 wt% of solid dosage can be completely consumed within 48 h of batch fermentation. Compared with other groups, the RCF hydrolysate obtained by the group with a 2-PrOH:H2O ratio of 5:5 (v/v) in the initial liquor exhibited the highest ethanol concentration of 25.49 ± 1.02 g/L, with a yield of 0.423 ± 0.017 g/g (of monomeric sugars in RCF hydrolysate). In contrast, due to the relatively lower monomer sugars concentration in hydrolysates of other groups, the ethanol concentration at the end of fermentations was lower than the best group, although similar yields and productivities can be achieved(Additional file 1: Table S9).

The ethanol fermentation performance of the 5:5 (v/v) group with and without nutrients addition was also analyzed, and the synthetic medium with similar glucose and xylose concentration was treated as the control. Ethanol production in the hydrolysate with nutrient addition was generally comparable to the control group, and slightly higher than the group without additional nutrients supplementation (Additional file 1: Table S10). However, considering the complexity of the fermentation process and the overall cost control, ethanol production using nutrient-absent RCF hydrolysate would be more feasible in realistic applications.

### Mass balance

**Fig. 5:**
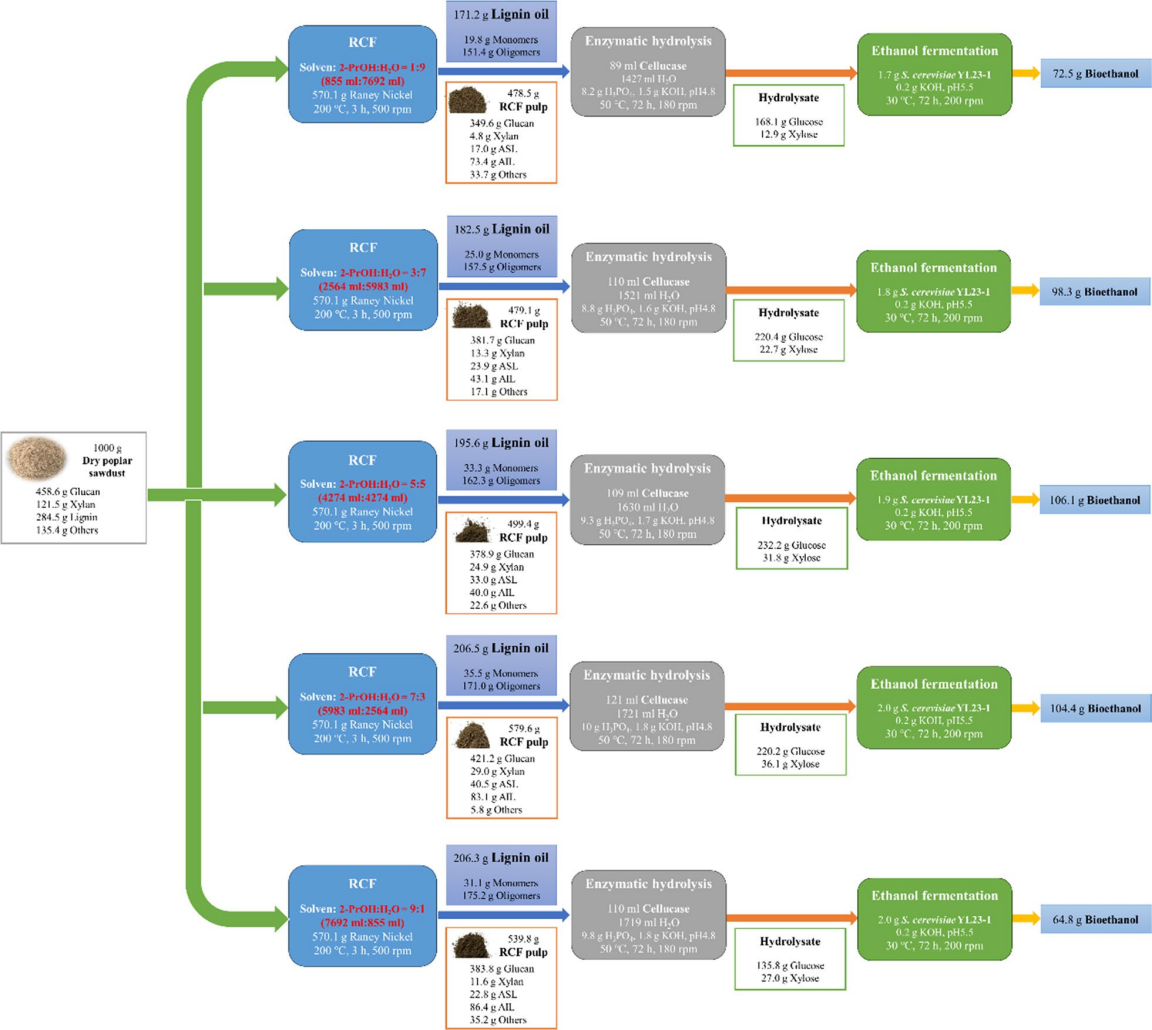
Mass balance of the sequential valorization of poplar sawdust into lignin oil and bioethanol using the initial liquor with different proportions

Mass balance of the biorefinery processes from RCF in terms of lignin oil and bioethanol co-generation is shown in Fig. 5. Generally, with an increase of 2-PrOH in the initial liquor, the yield of lignin oil gradually increased. The biorefinery process with a 2-PrOH:H_2_O ratio of 5:5 (v/v) in the initial liquor produced 195.6 g of lignin oil (which contained 33.3 g of lignin monomers), as well as 106.1 g of ethanol from 1000 g of poplar sawdust. In addition, despite a relatively low sugar concentration obtained from RCF pulp fractionated by the 2–7:3 (v/v) liquor, its higher solids recovery (579.6 g RCF pulp recovery from 1000 g poplar sawdust) ensured a high ethanol yield of 104.4 g from the raw material. In this group, 206.5 g of lignin oil (contained 35.5 g of lignin monomers) can be also co-generated from 1000 g of raw poplar sawdust.

## Conclusions

Poplar sawdust can be effectively valorized into well-defined lignin oil and bioethanol by integrating the hydrogen transfer RCF process with bioethanol fermentation. The 2-PrOH proportion in the initial liquor for the RCF could not only influenced the yield and constitution of the fractionated lignin oil, but also affected the structural features and chemical composition of the pulps. Results indicated that the initial liquor with the 2-PrOH:H_2_O ratio of 7:3 (v/v) provided a high lignin oil yield of 20.65 ± 0.31 wt%, and hydrogenation dominated reductive reactions in the RCF process. The monomeric sugars in hydrolysate of the RCF pulp can be converted into bioethanol by an engineered *S. cerevisiae* strain YL23-1 effectively. The mass balance showed that 206.5 g of lignin oil and 104.4 g of bioethanol can be co-generated from 1 kg of poplar sawdust based on the integrated biorefinery process. Overall, the outcome of this work provided an economical method for the effective fractionation and sequential utilization of all three major components (cellulose, hemicellulose and lignin) of poplar sawdust, showing promising in improving the economic feasibility of second-generation bioethanol production.

## Method and materials

### Raw material

Poplar sawdust was obtained from a local furniture factory in Nanjing, Jiangsu province of China. The contents of glucan, xylan, and lignin in the poplar sawdust were 45.86 ± 1.10 wt%, 12.15 ± 0.96 wt%, and 28.45 ± 1.21 wt%, respectively. Raney Ni (50 μm) catalyst was purchased from Aladdin Co., Ltd. Methanol (HPLC grade, ≥ 99.9 wt%), 2-PrOH (AR, ≥ 99.5 wt%) and DMSO-d6 (≥ 99.9 wt%) were purchased from Macklin Co., Ltd. Cellulase (Ctec 2, 145 ± 5 FPU/mL) was obtained from Novozymes Co., Ltd. Other chemicals under AR grade were purchased from Beijing Chemical Work.

### Reductive catalytic fractionation and hydrolysis of the pulp

RCF was conducted in a 250 mL of autoclave (Shanghai Yanzheng Experimental Instrument Co., Ltd.) with 100 mL of working volume. The initial liquor for RCF was a 2-PrOH:H_2_O mixture under different ratios (1:9, 3:7, 5:5, 7:3 and 9:1, v/v). For each batch of the RCF reaction, 11.70 g of poplar sawdust and 6.67 g of Raney Ni were mixed well with the 2-PrOH:H_2_O solution and the mixture was maintained at 200 °C for 3 h. The stirring rate was 500 rpm. After cooling to the room temperature (~ 25 °C), the slurry was separated by sequential magnet adsorption (to separate catalyst) and filtration (to separate the crude pulp). Then, the crude pulp was well-dispersed in anhydrous 2-PrOH at 500 rpm/min to separate the adhesive lignin oil remaining on the surface of pulp. After at least 20 batches of washing, the solid remaining was gathered and dried out in a vacuum, while the solution after washing was collected and combined with the reactant. Then, the mixture was evaporated in a vacuum at 45 °C until obtaining the dehydrated lignin oil [31].

Enzymatic hydrolysis of the RCF pulps was carried out in a 1 L of bioreactor (Shanghai Biotech Engineering Co. Ltd, Shanghai, China) with a 500 mL of working volume. Generally, the pulp was mixed with 0.05 M H_3_PO_4_/KH_2_PO_4_ buffer (adjusted pH to 4.8) at a solid to liquid ratio of 6 wt% or 12 wt%. The cellulase loading rate was 20 FPU/g (of glucan) [59]. After 72 h hydrolysis at 50 °C and 180 rpm, the liquid fraction was harvested by filtration and used as a substrate for bioethanol fermentation. Before the fermentation process, pH of the hydrolysates was adjusted to 4.8 by potassium hydroxide.

### The engineered strain and bioethanol fermentation

The strains used in this work are summarized in Additional file 1: Table S11. *Escherichia coli* Trans10 (TransGen Biotech) was used for the construction of the plasmid, and the culture medium consisted of 10 g/L peptone, 5 g/L yeast extract, 5 g/L NaCl. *S. cerevisiae* M3013 were laboratory stored and used as the parent strain for genetic manipulation [60]. The YP medium consisted of 20 g/L peptone, 10 g/L yeast extract, and 20 g/L glucose or xylose. The stock culture of *E. coli* and *S. cerevisiae* was preserved in 15% (v/v) glycerol at – 80 ℃.

The gene expression cassettes of the mXR-XDH pathway were obtained from previous work [56, 58]. *XUT4* and *XUT6* were isolated from *S. stipitis* CBS6054 with codon optimization in *S. cerevisiae* [61]. All genes were amplified by PrimeSTAR GXL DNA from TaKaRa. The primers, synthetic oligos and plasmids are constructed in Additional file 1: Table S11. The plasmids’ construction was confirmed by colony PCR using Taq RCR Master Mix from Biomed and sequencing. The GTR-CRISPR system with the donors and the related vectors pLacZ was constructed and co-transformed into *S. cerevisiae* by electroporation [62, 63]. Meanwhile, the genetic modification for strains construction was also confirmed by PCR amplification and sequencing. The successful deletion or the integration strains were cultured in G418-free medium for several generations to discard the plasmid pLacz containing the G418 screening marker.

ALE of *S. cerevisiae* YL23 was conducted in oxygen-limited 100 mL shake flasks containing 20 mL of YPX20 (20 g/L xylose). The engineered xylose-assimilating strain (name YL23) after ALE that with high xylose metabolism efficiency was screened and adopted for bioethanol fermentation using the RCF pulps’ hydrolysate. The fermentations were carried out without any other nutrients’ supplementation under oxygen limitation conditions in 250 mL shake flasks containing 50 mL substrate [64]. The inoculation rate of the YL23 seed was 10% (v/v, cultured in YPD medium, the OD_600_ was at ~ 1). Batch fermentation were carried out at 30 °C, 200 rpm for 48 h. All the above experiments were replicated in triplicate.

### Assay

The lignin monomers in the RCF lignin oils were qualitatively analyzed by GC–MS (Agilent 7890B GC and 5977B MS) that equipped with an HP-5 column (30 m × 0.25 mm). The detection conditions were as follows: temperatures for ion source and interface were 220 °C and 280 °C, respectively. The carrier gas flow rate was 1.2 mL/min and the injection volume was 2 μL. The detected spectra were referred to the NIST 11 library [18]. The monomer composition was quantified by external standard method using a gas chromatograph (GC-14C, Shimadzu, Japan) [31]. The molecular weight distribution of the lignin oil was determined by gel permeation chromatography (GPC, Waters 1525 gel chromatograph) that equipped with a differential refractive index detector and a Waters Styragel HT3/HT4/HT5 column at 35 °C. The interlinkage and the functional groups of the lignin fractions in the oil were tested using a Bruker AVANCE III 600M NMR spectrometer for ^1^H–^13^C HSQC NMR and quantitative phosphorus spectroscopy (^31^P-NMR). Data were integrated and semi-quantified using Bruker TopSpin 2.0 [65, 66].

Chemical composition of the RCF pulp was analyzed according to the standard of NREL [43]. Elemental analyses were carried out using an EL CUBE elemental analyzer (Elementar Analysensysteme GmbH, German). Micromorphology characterization of RCF pulps was conducted by an emission field scanning electron microscope (SEM, Hitachi SU8020) [24], and the X-ray diffraction spectroscopy (XRD) was analyzed by a Bruker D2 PHASER X-ray diffractometer.

The monomeric sugars concentration in enzymatic hydrolysates and fermentation broths were detected by high performance liquid chromatography (HPLC, Agilent Technologies 1200 Series, USA) equipped with an HPX-87P (Bio-Rad Labs, USA) column and a refractive index detector [64]. Bioethanol concentration was determined by gas chromatography (GC, TRACE1300, America) equipped with a glass packed column (Porapack Q, 80/100 mesh) and a flame ionization detector [67].

### Supplementary Information

**Additional file 1.** **Figure S1. **(A) Scatter plot of *δ*_*T*_ versus lignin monomers yields with different ratios of 2-PrOH:H_2_O (v/v) in initial liquor. (B) Scatter plot of *δ*_*T*_ versus delignification of pulps with different ratios of 2-PrOH:H_2_O (v/v) in initial liquor. (C) Scatter plot of RED versus lignin monomers yields with different ratios of 2-PrOH/H_2_O in initial liquor. (D) Scatter plot of RED versus delignification of pulps with different ratios of 2-PrOH/H_2_O in initial liquor. **Figure S2.** SEM images of RCF pulps using the pulping liquor with different 2-PrOH:H_2_O ratios. (A)-(F) represented to the raw poplar sawdust, and the pulps after RCF using the liquor that consisted of 1:9, 3:7, 5:5. 7:3 and 9:1 of 2-PrOH:H_2_O (v/v), respectively. **Figure S3. **Fermentation performances of the parent* S. cerevisiae* and engineered *S. cerevisiae* strains for ethanol production using the YPX medium containing 40 g/L of xylose. Time course of (A) OD_600_, (B) xylose, and (C) ethanol concentration in batch fermentation process. (D) Comparison of ethanol concentration, yield, and OD_600max_ in YPD medium using different strains. **Table S1. **Sugar concentrations after enzymatic hydrolysis of RCF pulps by different ratios of 2-PrOH:H_2_O (v/v). **Table S2.** Yield of RCF oil (wt%) using different ratios of 2-PrOH:H_2_O (v/v) as initial liquor, in which process 11.70 g dry poplar sawdust was mixture with the initial liquor for before fractionation. **Table S3.** Hildebrand solubility parameter (*δ*_*T*_) and HSP (*δ*_*D*_*, δ*_*P*_*, δ*_*H*_) for selected solvents (2-PrOH:H_2_O) and poplar lignin. **Table S4.** Molecular weight distribution of the lignin oil fractionated by different proportion of 2-PrOH:H_2_O (v/v) in the initial liquor. **Table S5.** Lignin monomers yield (wt%, lignin monomers yield=lignin monomers mass in the lignin oil / raw poplar sawdust × 100 %) using different proportion of 2-PrOH:H_2_O (v/v) in initial liquor. **Table S6.** Types and contents of hydroxyl groups in the RCF oil. **Table S7.** Crystallinity (CrI %) of the RCF pulps fractionated by different ratios of 2-PrOH:H_2_O. **Table S8.** Ethanol fermentation performances by parent* S. cerevisiae* and the engineered *S. cerevisiae* using YPX as substrate (40 g/L xylose). **Table S9.** Ethanol fermentation performances by *S. cerevisiae* YL23-1 using enzymatic hydrolysates of the RCF pulps. **Table S10**. Ethanol fermentation performances by *S. cerevisiae* YL23-1 using synthetic medium, enzymatic hydrolysates of the 5:5 of 2-PrOH:H_2_O (v/v) ratio group with and without nutrients (YP) addition. **Table S11.** *Saccharomyces cerevisiae*, plasmid, gRNA and primers used in this study.

## Data Availability

The data that support the findings of this study are available from the corresponding author upon reasonable request.
