# Supplementary material for: Sequential catalytic lignin valorization and bioethanol production: an integrated biorefinery strategy
Source: Biotechnol Biofuels Bioprod. 2024 Jan 20;17:8. doi: 10.1186/s13068-024-02459-8 (PMC10800047; doi:10.1186/s13068-024-02459-8)
Supplement: Supplementary file 1 — Additional file 1. Figure S1. (A) Scatter plot of δT versus lignin monomers yields with different ratios of 2-PrOH:H2O (v/v) in initial liquor. (B) Scatter plot of δT versus delignification of pulps with different ratios of 2-PrOH:H2O (v/v) in initial liquor. (C) Scatter plot of RED versus lignin monomers yields with different ratios of 2-PrOH/H2O in initial liquor. (D) Scatter plot of RED versus delignification of pulps with different ratios of 2-PrOH/H2O in initial liquor. Figure S2. SEM images of RCF pulps using the pulping liquor with different 2-PrOH:H2O ratios. (A)-(F) represented to the raw poplar sawdust, and the pulps after RCF using the liquor that consisted of 1:9, 3:7, 5:5. 7:3 and 9:1 of 2-PrOH:H2O (v/v), respectively. Figure S3. Fermentation performances of the parent S. cerevisiae and engineered S. cerevisiae strains for ethanol production using the YPX medium containing 40 g/L of xylose. Time course of (A) OD600, (B) xylose, and (C) ethanol concentration in batch fermentation process. (D) Comparison of ethanol concentration, yield, and OD600max in YPD medium using different strains. Table S1. Sugar concentrations after enzymatic hydrolysis of RCF pulps by different ratios of 2-PrOH:H2O (v/v). Table S2. Yield of RCF oil (wt%) using different ratios of 2-PrOH:H2O (v/v) as initial liquor, in which process 11.70 g dry poplar sawdust was mixture with the initial liquor for before fractionation. Table S3. Hildebrand solubility parameter (δT) and HSP (δD, δP, δH) for selected solvents (2-PrOH:H2O) and poplar lignin. Table S4. Molecular weight distribution of the lignin oil fractionated by different proportion of 2-PrOH:H2O (v/v) in the initial liquor. Table S5. Lignin monomers yield (wt%, lignin monomers yield=lignin monomers mass in the lignin oil / raw poplar sawdust × 100 %) using different proportion of 2-PrOH:H2O (v/v) in initial liquor. Table S6. Types and contents of hydroxyl groups in the RCF oil. Table S7. Crystallinity (CrI %) of the RCF pulps fra [file 13068_2024_2459_MOESM1_ESM.docx]

Sequential catalytic lignin valorization and bio-ethanol production: an integrated biorefinery strategy

Yilu Wu ^a^, Changsheng Su ^a^, Zicheng Liao ^b^, Gege Zhang ^c^, Yongjie Jiang ^a^, Yankun Wang ^a^, Changwei Zhang ^a^, Di Cai ^a,^*, Peiyong Qin ^b^, Tianwei Tan ^a^

^a^ National Energy R&D Center for Biorefinery, Beijing University of Chemical Technology, Beijing 100029, PR China

^b^ College of Life Science and Technology, Beijing University of Chemical Technology, Beijing 100029, PR China

^c^ School of International Education, Beijing University of Chemical Technology, Beijing 100029, PR China

Corresponding author

Email: caidibuct@163.com (D. Cai)


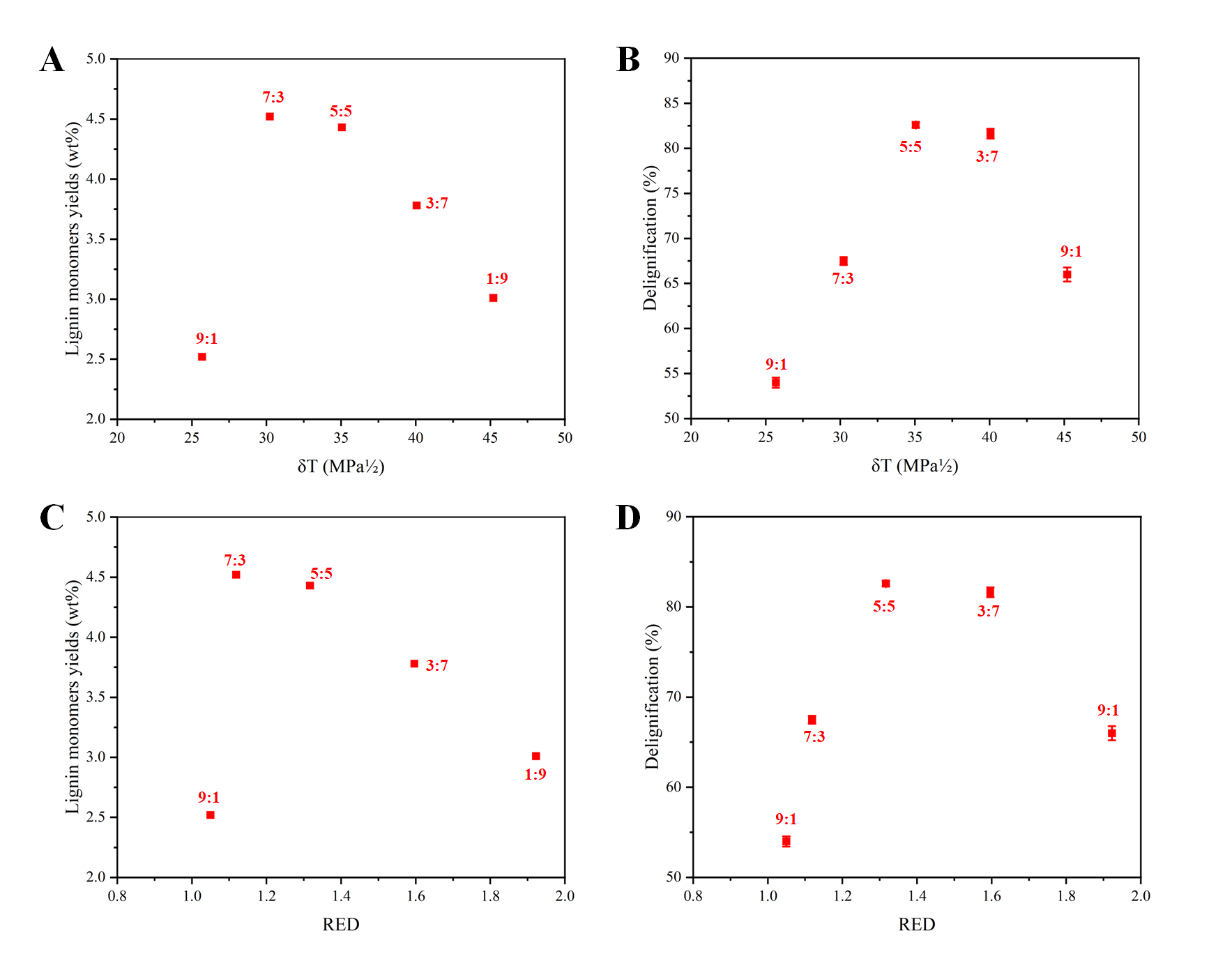


**Fig. S1** (A) Scatter plot of *δ_T_* versus lignin monomers yields with different ratios of 2-PrOH:H_2_O (v/v) in initial liquor. (B) Scatter plot of *δ_T_* versus delignification of pulps with different ratios of 2-PrOH:H_2_O (v/v) in initial liquor. (C) Scatter plot of RED versus lignin monomers yields with different ratios of 2-PrOH/H_2_O in initial liquor. (D) Scatter plot of RED versus delignification of pulps with different ratios of 2-PrOH/H_2_O in initial liquor.


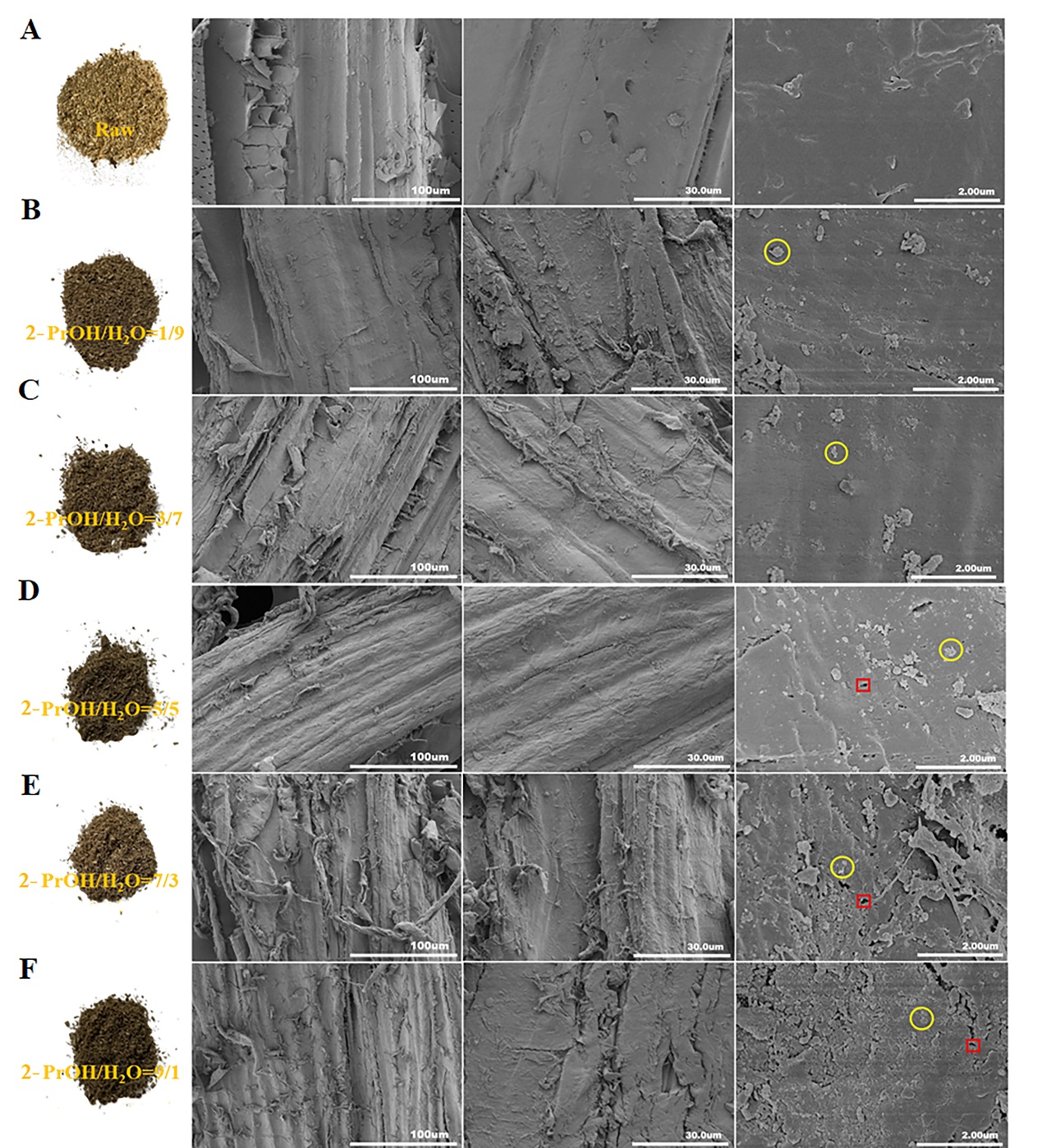


**Fig. S2** SEM images of RCF pulps using the pulping liquor with different 2-PrOH:H_2_O ratios. (A)-(F) represented to the raw poplar sawdust, and the pulps after RCF using the liquor that consisted of 1:9, 3:7, 5:5. 7:3 and 9:1 of 2-PrOH:H_2_O (v/v), respectively.


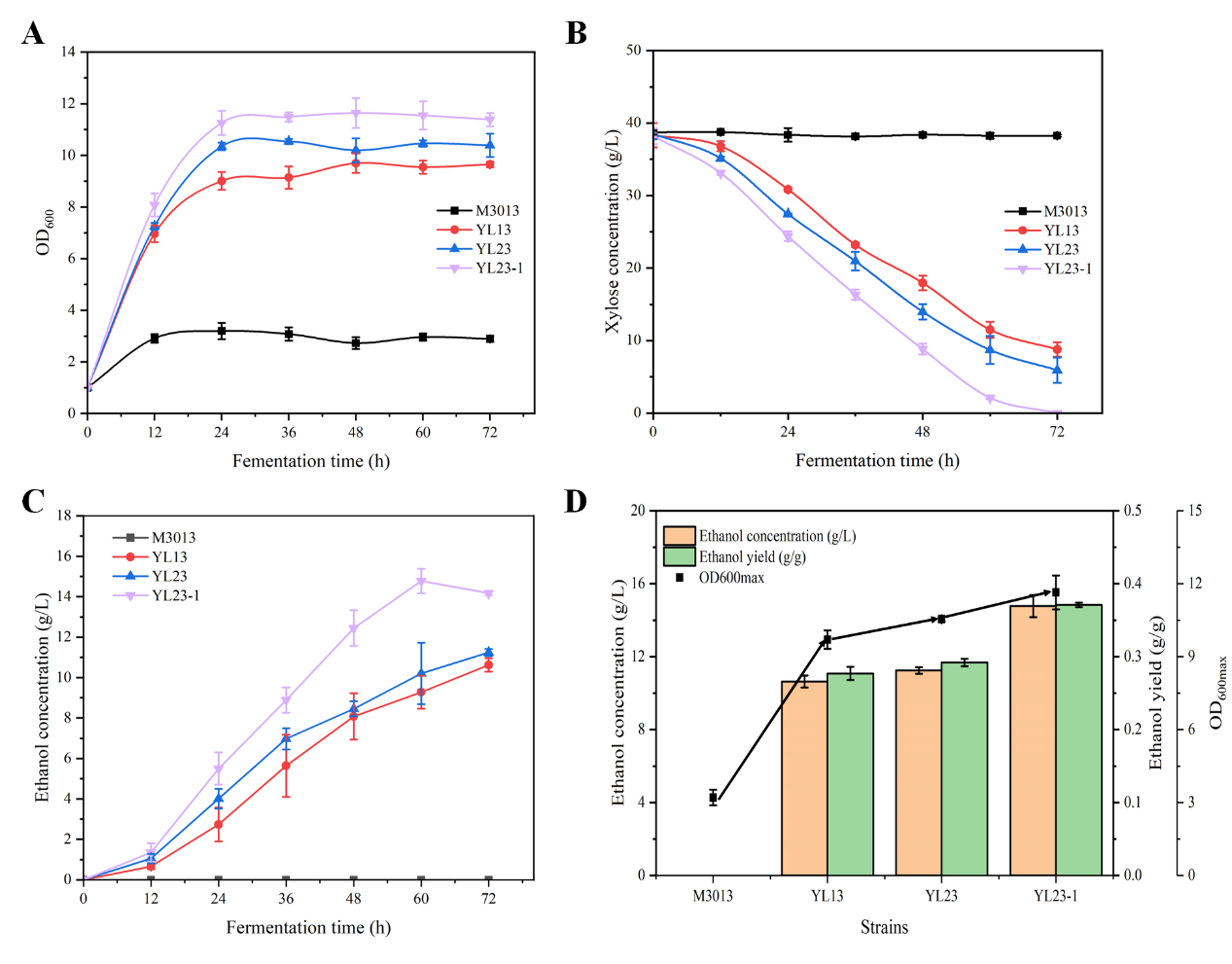


**Fig. S3** Fermentation performances of the parent *S. cerevisiae* and engineered *S. cerevisiae* strains for ethanol production using the YPX medium containing 40 g/L of xylose. Time course of (A) OD_600_, (B) xylose, and (C) ethanol concentration in batch fermentation process. (D) Comparison of ethanol concentration, yield, and OD_600max_ in YPD medium using different strains.

**Table S1** Sugar concentrations after enzymatic hydrolysis of RCF pulps by different ratios of 2-PrOH:H_2_O (v/v).

| 6 wt% solid dosage | Glucose concentration (g/L) | Xylose concentration (g/L) | Total sugar concentration (g/L) |
| --- | --- | --- | --- |
| 1:9 | 28.10±0.12 | 1.64±0.08 | 29.74±0.25 |
| 3:7 | 36.42±0.15 | 3.38±0.21 | 39.80±0.32 |
| 5:5 | 35.42±1.26 | 4.51±0.28 | 39.93±1.67 |
| 7:3 | 29.60±0.34 | 4.38±0.33 | 33.98±0.62 |
| 9:1 | 17.71±0.09 | 3.92±0.10 | 21.63±0.31 |
| 12 wt% solid dosage | Glucose concentration (g/L) | Xylose concentration (g/L) | otal sugar concentration (g/L) |
| 1:9 | 42.17±0.15 | 3.23±0.15 | 45.40±0.76 |
| 3:7 | 55.20±0.38 | 5.70±0.33 | 60.90±0.67 |
| 5:5 | 55.80±1.03 | 7.65±0.75 | 63.45±0.39 |
| 7:3 | 45.60±0.78 | 7.47±0.45 | 53.07±0.46 |
| 9:1 | 30.18±0.62 | 6.00±0.23 | 36.18±0.62 |

**Table S2** Yield of RCF oil (wt%) using different ratios of 2-PrOH:H_2_O (v/v) as initial liquor, in which process 11.70 g dry poplar sawdust was mixture with the initial liquor for before fractionation.

| **2-PrOH:H_2_O (v/v)** | **1:9** | **3:7** | **5:5** | **7:3** | **9:1** |
| --- | --- | --- | --- | --- | --- |
| **Mass (g)** | 2.003 ± 0.027 | 2.135 ± 0.020 | 2.289 ± 0.013 | 2.416 ± 0.020 | 2.420 ± 0.033 |
| **Yield (wt%)** | 17.12 ± 0.41 | 18.25 ± 0.33 | 19.56 ± 0.26 | 20.65 ± 0.31 | 20.63 ± 0.44 |

**Table S3** Hildebrand solubility parameter (*δ_T_*) and HSP (*δ_D_, δ_P_, δ_H_*) for selected solvents (2-PrOH:H_2_O) and poplar lignin.

|  | ***δ_D_* (MPa^½^)** | ***δ_P_* (MPa^½^)** | ***δ_H_* (MPa^½^)** | ***δ_T_* (MPa^½^)** | ***RED*** |
| --- | --- | --- | --- | --- | --- |
| **Lignin [1]** | 21.9 | 14.1 | 16.9 | 31.0 | / |
| **2-PrOH [2]** | 15.8 | 6.1 | 16.4 | 23.6 | 1.07 |
| **H_2_O [3]** | 15.5 | 16.0 | 42.3 | 47.8 | 2.10 |
| **1:9** | 15.5 | 15.0 | 39.7 | 45.2 | 1.92 |
| **3:7** | 15.6 | 13.0 | 34.5 | 40.1 | 1.60 |
| **5:5** | 15.7 | 11.1 | 29.4 | 35.0 | 1.32 |
| **7:3** | 15.7 | 9.1 | 24.2 | 30.2 | 1.12 |
| **9:1** | 15.8 | 7.1 | 19.0 | 25.7 | 1.05 |

The δ-values of aqueous solutions of selected solvents (2-PrOH:H_2_O) are calculated by *δ_M_* = *φ_1_δ_1_* + *φ_2_δ_2_*, where *φ* is the volume fraction of the 2-PrOH (index 1) or H_2_O (index 2), and *δ* is the *δ*-value of 2-PrOH (index 1) or H_2_O (index 2) [4].

*δ_T_^2^* = *δ_D_^2^*+*δ_P_^2^*+*δ_H_^2^*, *δ_T_* represented Hildebrand solubility parameter.

*RED*: Relative energy difference, was used to determine the miscibility of poplar lignin with selected solvents (2-PrOH:H_2_O). *RED* = *R_a_* / *R_0_*. *R_0_* is the radius of the Hansen sphere obtained by a computerized optimization method as described above. *R_a_^2^* = 4(*δ_D1_*-*δ_D2_*)^2^+(*δ_P1_*-*δ_P2_*)^2^+(*δ_H1_*-*δ_H2_*)^2^. The “1” referred to the selected solvents and “2” referred to lignin [2, 3].

**Table S4** Molecular weight distribution of the lignin oil fractionated by different proportion of 2-PrOH:H_2_O (v/v) in the initial liquor.

| **2-PrOH:H_2_O (v/v)** | ***Mn* (Da)** | ***Mw* (Da)** | **PDI** |
| --- | --- | --- | --- |
| 1:9 | 620 | 1050 | 1.694 |
| 3:7 | 681 | 1101 | 1.616 |
| 5:5 | 723 | 1282 | 1.773 |
| 7:3 | 724 | 1255 | 1.735 |
| 9:1 | 904 | 1114 | 1.233 |

**Table S5** Lignin monomers yield (wt%, lignin monomers yield=lignin monomers mass in the lignin oil / raw poplar sawdust × 100 %) using different proportion of 2-PrOH:H_2_O (v/v) in initial liquor.

| Types ^a^ | Lignin monomers | Lignin monomers yields (wt%) using different proportion of 2-PrOH:H_2_O (v/v) in initial liquor | | | | |
| --- | --- | --- | --- | --- | --- | --- |
|  |  | 1:9 | 3:7 | 5:5 | 7:3 | 9:1 |
| S | 4-(3-Hydroxypropyl)-2,6-dimethoxyphenol | 0.83 | 2.01 | 1.84 | 2.00 | 1.00 |
|  | 4-Propyl-2,6-dimethoxyphenol | 0.28 | 0.21 | 0.17 | 0.16 | 0.08 |
|  | 4-Ethyl-2,6-dimethoxyphenol | 0.00 | 0.00 | 0.00 | 0.00 | 0.00 |
|  | 4-Methyl-2,6-dimethoxyphenol | 0.07 | 0.01 | 0.25 | 0.35 | 0.04 |
|  | 2,6-Dimethoxyphenol | 0.02 | 0.03 | 0.01 | 0.02 | 0.04 |
| G | 4-(3-Hydroxypropyl)-2-methoxyphenol | 0.44 | 0.99 | 0.72 | 0.78 | 0.48 |
|  | 4-Propylguaiacol | 0.07 | 0.05 | 0.07 | 0.08 | 0.02 |
|  | 4-Ethyl-guaiacol | 0.26 | 0.13 | 0.30 | 0.29 | 0.11 |
|  | 4-Methylguaiacol | 0.07 | 0.07 | 0.03 | 0.03 | 0.01 |
| H | 4-(3-Hydroxypropyl)phenol | 0.83 | 0.07 | 0.74 | 0.67 | 0.62 |
|  | 4-Propylphenol | 0.07 | 0.13 | 0.07 | 0.06 | 0.03 |
|  | 4-Ethylphenol | 0.02 | 0.01 | 0.12 | 0.01 | 0.01 |
|  | 4-Methylphenol | 0.00 | 0.00 | 0.00 | 0.00 | 0.00 |
| Others | 4-Propylcyclohexanol | 0.01 | 0.05 | 0.08 | 0.02 | 0.04 |
|  | 4-Ethylcyclohexanol | 0.04 | 0.02 | 0.03 | 0.05 | 0.04 |
|  | 4-Methylcyclohexanol | 0.00 | 0.00 | 0.00 | 0.00 | 0.00 |
|  | Total | 3.01 | 3.78 | 4.43 | 4.52 | 2.52 |

^a^ S: Syringl units; G: Guaiacyl units; H: *p*-Hydroxyphenyl units.

**Table S6** Types and contents of hydroxyl groups in the RCF oil.

| **2-PrOH:H_2_O ratios (v/v)** | **S-OH**  **(mmol/g)** | **G-OH**  **(mmol/g)** | **H-OH**  **(mmol/g)** | **Phenolic-OH**  **(mmol/g)** | **Aliphatic-OH**  **(mmol/g)** | **COOH**  **(mmol/g)** |
| --- | --- | --- | --- | --- | --- | --- |
| **1:9** | 0.413 | 0.111 | 0.467 | 1.991 | 21.580 | 4.203 |
| **3:7** | 0.070 | 1.104 | 0.007 | 1.181 | 28.601 | 3.871 |
| **5:5** | 0.420 | 1.192 | 0.458 | 2.070 | 29.860 | 4.268 |
| **7:3** | 0.229 | 0.845 | 0.279 | 1.353 | 31.724 | 4.827 |
| **9:1** | 0.081 | 1.018 | 0.222 | 1.321 | 13.752 | 4.806 |

**Table S7** Crystallinity (CrI %) of the RCF pulps fractionated by different ratios of 2-PrOH:H_2_O.

| **2-PrOH:H_2_O (v/v)** | **CrI (%)** |
| --- | --- |
| **1:9** | 51.42 |
| **3:7** | 51.38 |
| **5:5** | 53.60 |
| **7:3** | 66.50 |
| **9:1** | 63.72 |

**Table S8** Ethanol fermentation performances by parent *S. cerevisiae* and the engineered *S. cerevisiae* using YPX as substrate (40 g/L xylose).

| **Strain** | **M3013** | **YL13** | **YL23** | **YL23-1** |
| --- | --- | --- | --- | --- |
| **Residual xylose (g/L)** | 38.25±0.18 | 8.77±1.01 | 5.91±1.74 | 0±0.00 |
| **OD_600max_** | 3.20±0.32 | 9.70±0.38 | 10.54±0.10 | 11.64±0.70 |
| **Ethanol concentration (g/L)** | 0±0.00 | 10.63±0.33 | 11.24±0.18 | 14.77±0.60 |
| **Ethanol yield (g/g)** | 0±0.00 | 0.277±0.009 | 0.292±0.005 | 0.371±0.003 |

**Table S9** Ethanol fermentation performances by *S. cerevisiae* YL23-1 using enzymatic hydrolysates of the RCF pulps.

| **2-PrOH:H_2_O (v/v)** | **1:9** | **3:7** | **5:5** | **7:3** | **9:1** |
| --- | --- | --- | --- | --- | --- |
| **Ethanol concentration (g/L)** | 18.19±1.02 | 24.63±1.05 | 25.49±1.02 | 21.61±0.62 | 14.38±0.90 |
| **Ethanol yield (g/g)** | 0.422±0.023 | 0.426±0.018 | 0.423±0.017 | 0.429±0.010 | 0.418±0.026 |

**Table S10** Ethanol fermentation performances by *S. cerevisiae* YL23-1 using synthetic medium, enzymatic hydrolysates of the 5:5 of 2-PrOH:H_2_O (v/v) ratio group with and without nutrients (YP) addition.

| **Medium** | **Synthetic medium** | **Enzymatic hydrolysate with nutrients addition** | **Enzymatic hydrolysate without nutrients addition** |
| --- | --- | --- | --- |
| **Ethanol concentration (g/L)** | 26.38±0.48 | 26.12±0.88 | 25.49±1.02 |
| **Ethanol yield (g/g)** | 0.434±0.08 | 0.431±0.015 | 0.423±0.017 |

**Table S11** *Saccharomyces cerevisiae*, plasmid, gRNA and primers used in this study.

| **Strain** | **Relevant Genotype and Manipulation** | | **Source** |
| --- | --- | --- | --- |
| M3013 | Parent strain used for bioethanol fermentation. | | Laboratory preservation |
| YL13 | M3013 derivative, XI-3:: P*_TEF2_-****XYL1***, P*_TEF1_-****mXYL1***, P*_PGK1p_-****XYL2***, P_HXT7p_*-****XKS1***, ***PHO13***::gRNA, ***GRE3***::gRNA, XII-2:: P*_TDH1p_-****RPE1***, P*_PGK1p_-****TAL1***, P*_TEF1p_-****RKI1***, P*_TDH3p_-****TKL1*** | |  |
| YL23 | YL13 derivative, XI-5:: P*_PGK1_-****XUT4***, *P_PGK1_-****XUT6*** | | This work |
| YL23-1 | YL23 derivative, single-colony isolate from adaptive laboratory evolution by xylose | | This work |
| **Plasmid** | **Relevant properties or genotype** | | **Source** |
| pRS03 | pSPGM1 carrying *XUT4* and *XUT6* | | This work |
| pLacZ-XII-5 | ori, AmpR, 2μ, G418, pTEF1-iCas9-tADH1, pSNR52-(XII-5 gRNA)-gRNA scaffold-tSNR52  **20bp Target Sequence**: CTATAACCGGTTTGAATTTA, TTGTCACAGTGTCACATCAG | | This work |
| **Primer** | | **Nucleotide sequence (5’ >3’)** | |
| P-XUT4-F | | CGCGGATCCGCGTTCCATCtCATTCAACTTGTACTTAAAGAT | |
| P-XUT4-R | | TCCCCGCGGGGAATGTCTTCGTTATTGACTAACGAATACTT | |
| XUT4-YAN-F | | TGTGACAACAACAGCCTGTTCTCA | |
| XUT4-YAN-R | | TTGCTTTTATCTGGATCTGTGCTCA | |
| P-XUT6-F | | TCCCACTAGTGGGAATGTCCAGTGTTGAAAAAAGTGCT | |
| P-XUT6-R | | CGGGAGCTCCCGTTAGCTGATGTTTTCGACATGCTCT | |
| XUT6-YAN-F | | GTGCGGGCCTCTTCGCTA | |
| XUT6-YAN-R | | CCTGGTTAGGTGGATCCTGGT | |
| Donor-XUT4-F | | GATGAGTCACTGACAGCCACCGCAGAGGTTCTGACTCCTACTGAGCTCTATACGACTCACTATAGGGCGAATTGGGTACC | |
| Donor-XUT6-R | | CTGTCACTGAACTAAAACAATAAGGCTAGTTCGAATGATGAACTTGCTTGATCAAGCTTATCGATACCGTCGACCTCGAG | |
| Goden-XII-5-F | | AAAGGTCTCTGATCTTGTCACAGTGTCACATCAGGTTTTAGAGCTAGAAATAGCAAGTTAAAATAAGGC | |
| Goden-XII-5-R | | AAAGGTCTCTAAACTAAATTCAAACCGGTTATAGGATCATTTATCTTTCACTGCGGAGAAGT | |
| Cas9-I-F | | CGATTTTTGTGATGCTCGTCAGGG | |
| Cas9-I-R | | AAAAAGAAGAGAAAGGTCTGACTCGAG | |

**References**

[1] K. Huang, M. Mohan, A. George, B.A. Simmons, Y. Xu, J.M. Gladden, Integration of acetic acid catalysis with one-pot protic ionic liquid configuration to achieve high-efficient biorefinery of poplar biomass, Green Chemistry. 2021; 23(16): 6036-6049. <https://doi.org/10.1039/d1gc01727f>.

[2] S.R. Haiyue Ni, G. Fang, Y. Ma, Determination of Alkali Lignin Solubility Parameters by Inverse Gas Chromatography and Hansen Solubility Parameters, BioResources. 2016; 11(2): 4353-4368.

[3] R. Rinken, D. Posthuma, R. Rinaldi, Lignin Stabilization and Carbohydrate Nature in H‐transfer Reductive Catalytic Fractionation: The Role of Solvent Fractionation of Lignin Oil in Structural Profiling**, ChemSusChem. 2022; 16(3). <https://doi.org/10.1002/cssc.202201875>.

[4] F. Cheng, T. Ouyang, J. Sun, T. Jiang, J. Luo, Using Solubility Parameter Analysis to Understand Delignification of Poplar and Rice Straw with Catalyzed Organosolv Fractionation Processes, BioResources. 2019; 14(1): 486-499.
